# Challenges and Future Directions in Lipoprotein Measurement for Atherosclerosis Prevention and Treatment

**DOI:** 10.3390/ijms252413247

**Published:** 2024-12-10

**Authors:** Tomokazu Konishi

**Affiliations:** Graduate School of Bioresource Sciences, Akita Prefectural University, Akita 010-0195, Japan; konishi@akita-pu.ac.jp

Atherosclerosis can cause severe damage to the heart, brain, and other vital organs. In particular, cardiovascular diseases are crucial to consider, especially as their prevalence increases with age [1]. Abnormalities in lipoproteins are a major contributing factor to this condition [2]. As there are many classes of lipoproteins [3], each with distinct functions, it is essential to measure these classes accurately.

Since the 1950s, isodensity centrifugation has been employed for the biochemical separation of lipoprotein classes [4,5,6,7]. However, due to its time-consuming and labor-intensive nature, simplified methods yielding similar results have been developed and are widely used in medical examinations [8].

Nevertheless, recent findings have highlighted a variety of issues with these methods. High-performance liquid chromatography (HPLC) separation by gel filtration has revealed the existence of at least 12 distinct classes of human lipoproteins, and these cannot be adequately separated by ultracentrifugation [3,9]. Moreover, some of these classes have been unequivocally associated with various diseases [2]. This level of detail cannot be achieved through isodensity centrifugation or its related simplified methods [10,11]. Notably, the HDL and LDL measured by these simplified methods do not correspond to the actual classes identified [3], and the ultracentrifugal method tends to overestimate HDL levels [3].

How did this discrepancy arise? This may be attributed to the peculiarities of the isodensity centrifugation method. This technique involves a process whereby a salt concentration gradient is created by applying strong centrifugal force for an extended period at very high salt concentrations, primarily using sodium and potassium chloride. The differences in specific gravity are then used to separate lipoproteins with varying densities [4,5,6,7].

Each protein has its own specific water solubility level [12]. Many protein components of lipoproteins contain both hydrophobic and hydrophilic regions, yet lipoproteins as a whole are water soluble. If they were not, they would block blood vessels. However, proteins exhibit a phenomenon known as “salting out” [13]. This refers to the addition of salt at a certain concentration level, depending on the protein type, which causes the protein to become insoluble in water, leading to precipitation. This principle is the basis of a fundamental technique often used in biochemical protein isolation. Conversely, some proteins become more soluble when salt is added, a process known as “salt solubilization”. There is a close relationship between salt concentration and protein solubility, with the salt concentration at which a protein becomes soluble varying for each specific protein.

Ultracentrifugation inevitably employs very high salt concentrations [4,5,6,7] and is not typically used for complexes with a large number of proteins. Isodensity centrifugation is a rather unusual technique usually used to separate more solid molecules, such as DNA or viral particles. When separating using density gradients, these gradients are often prepared in advance—for example, by using substances with little or no ionic strength, such as sucrose, or polymers such as silica sols [14]. This approach helps avoid saltation and allows for the desired density gradient to be achieved at a much faster rate than with self-gradient autophoresis.

Accordingly, it was presumed that the isodensity centrifugation method would destroy many complex lipoproteins. In particular, large classes such as chylomicrons (CMs) and very-low-density lipoproteins (VLDLs) would have lost most of their proteins due to salting out, which is not suitable when separating such complexes. It was therefore believed that these classes contained little protein and were almost entirely composed of lipids [12,15]. However, this would not have allowed for the stable retention of lipids in the blood. In fact, the predominant component of these classes separated by HPLC was not lipids but proteins; there is likely little difference in the specific density between the lipoprotein classes [6]. This is likely the reason the more common method of ultracentrifugation, using a pre-prepared density gradient, was not employed; separation might not have been achieved without destroying lipoproteins. Incidentally, the class names follow the previous ones for the purposes of avoiding confusion, although perhaps HDL is not as dense by comparison [6].

This led to the fraction considered to be HDL in isodensity centrifugation appearing much larger than it actually was [3]. The fraction thought to be very dense was probably a degraded product of other classes of salted-out lipoproteins—their remnants rather than the HDLs themselves [3]. This resulted in another significant error. Naturally, the remnants that were salted out contained more proteins and were denser.

When lipoproteins are separated by HPLC, two large peaks prominently stand out (Figure 1). This observation led to misinterpretation, partly due to the results of isodensity centrifugation suggesting a high concentration of HDL and partly due to the visual appearance of the peaks. These peaks were simply interpreted as LDL and HDL [16,17,18]. However, the second peak is actually large in diameter, more than 10 nm. Given that HDLs have a protein-covered outer structure that limits the size of their diameter, they cannot be this large [19]. The HPLC data analysis was thus distorted to align with the results of isodensity ultracentrifugation [18,20]. In reality, this second peak is predominantly composed of LDL2 and LAC2, the cholesterol-distributing classes [3]. The original HDLs are, in fact, very minor in human blood (Figure 1).

This interpretation of the HPLC results was initially unclear until the data were analyzed using a data-driven approach, which eliminated assumptions such as the abundance of HDL [9]. Several methods based on various assumptions were proposed [16,17,18], but all of these appeared to be incorrect. Ultimately, the results could not be interpreted until the protein components of the fractions were identified and data with minimal parameters were explained (Figure 1) [3,9]. Although a minimum of 12 distinct classes were considered for human samples [3], future studies will likely expand this number.

A similar issue arises in cases of Nuclear Magnetic Resonance (NMR) measurements [21]. NMR is a technique used to study the atomic states of nuclei in a magnetic field by analyzing their interaction with electromagnetic waves at specific frequencies. While spectra for relatively small molecules can easily be interpreted, the measurement and analysis of more complex molecules, such as proteins, are significantly more challenging and time consuming, particularly for samples that have not been isotope labeled. For lipoproteins, which are large complexes of lipids and proteins, accurate measurement is nearly impossible without correlating NMR data with results from other techniques. Consequently, NMR measurements often rely on the assumption that they align with isodensity centrifugation results. By contrast, HPLC allows the eluted sample to be aliquoted and analyzed biochemically (this technique is often referred to as Fast Protein Liquid Chromatography) if needed, offering the advantage of more reliable signal attribution. Although several alternative methods including NMR have been developed [22], they all depend on the same assumption of agreement with isodensity centrifugation, which compromises the accuracy of their results, similar to enzymatic methods [3,8]. It is possible that this issue could be resolved by using and retraining HPLC data. Anyway, due to the practical advantages of enzyme-based methods, these alternatives have not gained widespread acceptance in the medical community at present.

Given the significant errors, it appears inevitable that LDL and HDL, especially as measured by simpler methods, do not accurately reflect the true health status. These metrics represent a composite of multiple classes, which poses a serious issue considering the vast number of studies that have relied on such data. In practice, however, it has been observed that measurements of LDL, for instance, show no correlation with prognosis, even in large cohort studies [10,11].

In fact, several classes measured by HPLC are strongly suspected to be involved in disease [2]. For example, elevated LCA1 and reduced HDL1 are significant risk factors. By contrast, our data showed no risk associated with LDL. While these data might be somewhat biased (as our region is known for a high prevalence of atherosclerosis), it is clear that lowering LDL levels through statin use did not prevent the progression of atherosclerosis, at least based on our data.

From this, the importance of precision in analyzing data becomes evident. It suggests that traditional methods, such as isodensity centrifugation [4,5,6,7] and its simplified variants [8], can no longer be relied upon. Clearly, new methods are required. While HPLC is a highly effective albeit labor-intensive method, it is not suitable for processing large numbers of samples. Therefore, an automated method, perhaps using antibodies or enzymes, is necessary. If only a few classes were accurately identified, the underlying problem for each individual would become clearer, potentially leading to the development of new drugs or treatments.

For instance, if electrophoresis could be used to separate samples, it would be more suitable for multiple samples than HPLC. Although many methods have been used for serum electrophoresis, none can discriminate between lipoprotein classes. Therefore, a new system is required. Additionally, as with HPLC, a high level of analysis may be necessary to quantify the data, which would still constitute a barrier to measuring multiple samples, even though AI could potentially assist in this process.

A more practical solution involves measuring and quantifying specific classes of reactions that can be automated, similar to current methodologies for colorimetric or turbidity measurements, which are commonly employed in various health assessments. If antibodies against specific proteins in the class can be produced, techniques such as immunoturbidimetry or nephelometry could be utilized. This would be a similar approach to that used in the measurement of Lp(A) [23]. However, it is important to note that these methods only quantify the protein content, which does not necessarily correlate with cholesterol levels within the class. Measuring cholesterol presents a more complex challenge, as the amount of protein is not always proportional to cholesterol concentration. Alternatively, if the enzyme responsible for producing the class is identified, its quantity may be indirectly estimated through enzyme activity measurements. Additionally, if only certain classes can be solubilized, or if cholesterol can be selectively expelled using class-specific receptors, these approaches could yield the most accurate measurements.

## Figures and Tables

**Figure 1:**
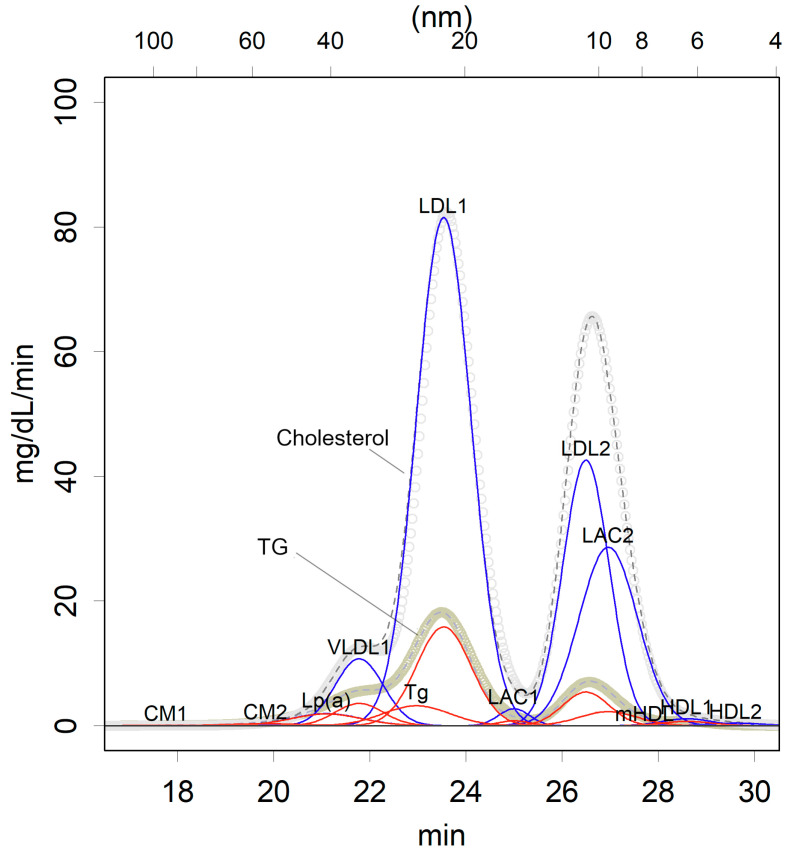
An example of an HPLC profile of healthy lipoproteins, which was used in a previously mentioned article [3]. Molecules elute from the gel in order of increasing diameter (x-axis, from left to right). The elution time is proportional to the logarithm of the molecule’s diameter (displayed above the x-axis). The triglycerides (TGs) and cholesterol concentrations in the eluted solution are measured and recorded over time (gray). These measurements are approximated using the fewest number of normal distributions (red: TG; blue: cholesterol). The rationale for using a normal distribution is based on the behavior of molecules passing through the column. When a molecule of a certain size moves through the column, it randomly enters and exits the gel matrix, resulting in a normally distributed elution time due to the Central Limit Theorem. For instance, glycerol and plastic beads exhibit normal distribution in their elution times. The elution times for specific classes of TG and cholesterol are common. The standard deviation (σ) differs slightly between each class, indicating the degree of heterogeneity within the population of each class. Under these conditions, at least 12 distinct classes are required. Each class is identified by the detection of specific proteins. Notably, there are two prominent peaks, both of which correspond to LDL and its derivatives, while HDL is represented by much smaller peaks.

## Data Availability

The sample data are available in figushare; https://doi.org/10.6084/m9.figshare.14247119.v1 (accessed 9 December 2024).

## References

[1] Rodgers J.L., Jones J., Bolleddu S.I., Vanthenapalli S., Rodgers L.E., Shah K., Karia K., Panguluri S.K. (2019). Cardiovascular Risks Associated with Gender and Aging. J. Cardiovasc. Dev. Dis..

[2] Konishi T., Hayashi Y., Fujiwara R., Kawata S., Ishikawa T. (2023). Distinctive features of lipoprotein profiles in stroke patients. PLoS ONE.

[3] Konishi T., Fujiwara R., Saito T., Satou N., Crofts N., Iwasaki I., Abe Y., Kawata S., Ishikawa T. (2022). Human lipoproteins comprise at least 12 different classes that are lognormally distributed. PLoS ONE.

[4] Havel R.J., Eder H.A., Bragdon J.H. (1955). The distribution and chemical composition of ultracentrifugally separated lipoproteins in human serum. J. Clin. Investig..

[5] Redgrave T.G., Roberts D.C., West C.E. (1975). Separation of plasma lipoproteins by density-gradient ultracentrifugation. Anal. Biochem..

[6] Chapman M.J., Goldstein S., Lagrange D., Laplaud P.M. (1981). A density gradient ultracentrifugal procedure for the isolation of the major lipoprotein classes from human serum. J. Lipid Res..

[7] Nakamura M., Kayamori Y., Iso H., Kitamura A., Kiyama M., Koyama I., Nishimura K., Nakai M., Noda H., Dasti M. (2014). LDL cholesterol performance of beta quantification reference measurement procedure. Clin. Chim. Acta.

[8] Miller W.G., Myers G.L., Sakurabayashi I., Bachmann L.M., Caudill S.P., Dziekonski A., Edwards S., Kimberly M.M., Korzun W.J., Leary E.T. (2010). Seven direct methods for measuring HDL and LDL cholesterol compared with ultracentrifugation reference measurement procedures. Clin. Chem..

[9] Konishi T., Takahashi Y. (2018). Lipoproteins comprise at least 10 different classes in rats, each of which contains a unique set of proteins as the primary component. PLoS ONE.

[10] Grundy S.M., Stone N.J., Bailey A.L., Beam C., Birtcher K.K., Blumenthal R.S., Braun L.T., Ferranti S.d., Faiella-Tommasino J., Forman D.E. (2019). 2018 AHA/ACC/AACVPR/AAPA/ABC/ACPM/ADA/AGS/APhA/ASPC/NLA/PCNA Guideline on the Management of Blood Cholesterol: A Report of the American College of Cardiology/American Heart Association Task Force on Clinical Practice Guidelines. Circulation.

[11] Robinson J.G., Stone N.J. (2015). The 2013 ACC/AHA guideline on the treatment of blood cholesterol to reduce atherosclerotic cardiovascular disease risk: A new paradigm supported by more evidence. Eur. Heart J..

[12] Voet D., Voet J.G. (2004). Biochemistry.

[13] Moringo N.A., Bishop L.D.C., Shen H., Misiura A., Carrejo N.C., Baiyasi R., Wang W., Ye F., Robinson J.T., Landes C.F. (2019). A mechanistic examination of salting out in protein-polymer membrane interactions. Proc. Natl. Acad. Sci. USA.

[14] Pertoft H. (2000). Fractionation of cells and subcellular particles with Percoll. J. Biochem. Biophys. Methods.

[15] Conn E.E., Stumpf P.K., Bruening G., Doi R.H. (1987). Outlines of Biochemistry.

[16] Okazaki M., Usui S., Ishigami M., Sakai N., Nakamura T., Matsuzawa Y., Yamashita S. (2005). Identification of unique lipoprotein subclasses for visceral obesity by component analysis of cholesterol profile in high-performance liquid chromatography. Arterioscler. Thromb. Vasc. Biol..

[17] Okazaki M., Yamashita S. (2016). Recent advances in analytical methods on lipoprotein subclasses: Calculation of particle numbers from lipid levels by gel permeation HPLC using “spherical particle model”. J. Oleo Sci..

[18] Gordon S.M., Deng J., Lu L.J., Davidson W.S. (2010). Proteomic characterization of human plasma high density lipoprotein fractionated by gel filtration chromatography. J. Proteome Res..

[19] Wu Z., Gogonea V., Lee X., May R.P., Pipich V., Wagner M.A., Undurti A., Tallant T.C., Baleanu-Gogonea C., Charlton F. (2011). The low resolution structure of ApoA1 in spherical high density lipoprotein revealed by small angle neutron scattering. J. Biol. Chem..

[20] Suto A., Yamasaki M., Takasaki Y., Fujita Y., Abe R., Shimizu H., Ohta H., Takiguchi M. (2013). LC-MS/MS analysis of canine lipoproteins fractionated using the ultracentrifugation-precipitation method. J. Vet. Med. Sci..

[21] Baumstark D., Kremer W., Boettcher A., Schreier C., Sander P., Schmitz G., Kirchhoefer R., Huber F., Kalbitzer H.R. (2019). 1H NMR spectroscopy quantifies visibility of lipoproteins, subclasses, and lipids at varied temperatures and pressures. J. Lipid Res..

[22] Islam S.M.T., Osa-Andrews B., Jones P.M., Muthukumar A.R., Hashim I., Cao J. (2022). Methods of Low-Density Lipoprotein-Cholesterol Measurement: Analytical and Clinical Applications. EJIFCC.

[23] Heydari M., Rezayi M., Ruscica M., Jamialahmadi T., Johnston T.P., Sahebkar A. (2023). The ins and outs of lipoprotein(a) assay methods. Arch. Med. Sci.–Atheroscler. Dis..

